# Efficacy of lufenuron on *Allium cepa*: cytogenotoxic, molecular and *in silico *docking studies

**DOI:** 10.1007/s10646-026-03048-1

**Published:** 2026-02-18

**Authors:** Yudum Yeltekin Uğur, Recep Liman, Rahsan Ilikci-Sagkan

**Affiliations:** 1 Molecular Biology and Genetics Department, Faculty of Engineering and Natural Sciences, Uşak University, Uşak, 64300 Türkiye; 2 Medical Biology Department, School of Medicine, Uşak University, Uşak, 64300 Türkiye

**Keywords:** Allium test, Apoptosis, Chromosome aberrations, DNA damage, Flow cytometer, RAPD

## Abstract

Lufenuron, a benzoylurea insecticide that inhibits chitin synthesis, was evaluated for its cytogenotoxic and DNA-damaging effects on *Allium cepa* root meristem cells using root growth inhibition, Allium anaphase-telophase, comet, RAPD-PCR, and flow cytometry assays. Molecular docking was also performed to investigate the interactions of lufenuron with cytochrome P450 enzymes (CYP81A12 and CYP81A21) and a standard B-form double-stranded DNA dodecamer model (12 base pairs). Root growth significantly decreased from 3.87 ± 0.44 cm at low doses to 0.26 ± 0.12 cm at high concentrations according to the root growth inhibition test. Lufenuron exposure caused dose- and time-dependent reductions in mitotic index (MI) and increases in chromosomal aberrations (CAs) in anaphase-telophase cells such as disturbed anaphase-telophase, chromosomal laggards, stickiness, anaphase bridges) and other cells (micronuclues and c-metaphase) along with DNA damage. The results of flow cytometry demonstrated the existence of subG1 arrest-induced early apoptosis. Compared with the negative control, lufenuron-treated roots exhibited distinct RAPD polymorphisms characterized by the loss and/or emergence of specific DNA bands. Numerical and phenetic analyses of RAPD profiles clearly demonstrated a dose- and time-dependent genotoxic response to lufenuron exposure. Molecular docking studies showed that lufenuron binds strongly to CYP81A12, CYP81A21, and DNA with greater affinity than the positive control (MMS), fitting into enzyme active sites and the DNA minor groove with an intercalation gap. Overall, the study demonstrates that lufenuron exerts notable cytogenotoxic and DNA-damaging effects on *A. cepa* roots. Therefore, its agricultural use should be carefully regulated to minimize potential risks to non-target organisms and environmental health.

## Introduction

Insect growth regulators (IGRs) such as lufenuron are a class of insecticides that disrupt the physiological development of insects by inhibiting processes such as molting, new exoskeleton formation, and feeding, ultimately leading to pest mortality. Unlike traditional insecticides that target the nervous system, IGRs have a unique mode of action, resulting in lower toxicity to non-target organisms, reduced environmental pollution, and minimal impact on natural enemies and beneficial species. These attributes make IGRs valuable tools in sustainable agriculture, supporting the production of eco-friendly, residue-free food and promoting human health. Consequently, IGRs are often referred to as “third-generation pesticides”, “pesticides of the 21st century”, “bio-regulators”, and “novel materials for insect control” (Clifton and Lopez 2025; Gad et al. 2021; Rui et al. 2012; Sankar and Kumar 2023; Tunaz and Uygun 2004).

Lufenuron, (RS)-1-[2,5-dichloro-4-(1,1,2,3,3,3-hexafluoropropoxy)phenyl]-3-(2,6-difluorobenzoyl)urea, is a benzoylurea-class chitin synthesis inhibitor (El-Sheikh 2015; Lv et al. 2022). It exhibits strong ovicidal properties and adversely affects the digestive systems of target pests such as *Spodoptera littoralis* and *S. exiqua* in general crops of cotton, corn, cucumber and pepper in Turkiye, *Aphis craccivora*,* Frankliniella occidentalis*, *Bactrocera dorsalis* and *Phyllocoptruta oleivora* in citrus orchards and *S. frugiperda* in maize and cotton in other countries (Lu et al. 2023; Xia et al. 2024). It was shown that lufenuron exhibited high insecticidal activity against *S. frugiperda*, significantly prolonging larval developmental duration and reducing pupation and emergence rates by reducing the expression of the chitinase gene while maintaining relatively stable expression levels of chitin synthase (Lv et al. 2022). Yang et al. (2016) found that lufenuron treatment may also influence miRNA expression (miR-71 and miR-263), thereby affecting the regulation of chitin synthase and chitinase genes.

Although lufenuron acts selectively on target species, there is evidence suggesting the potential for bioaccumulation and detrimental consequences on non-target organisms. Ingestion of food residues, inhalation, or skin contact all increase exposure to pesticides when their use and application rise. Lufenuron induced reproductive toxicity and genotoxic effects in pregnant albino rats and their fetuses (Basal et al. 2020), mortality rate and toxic effects in fish of *Colossoma macropomum* (Soares et al. 2016), biochemical and histological alterations in the midgut epithelium of *Podisus nigrispinus* (Lira et al. 2020), and also decreased growth rate of earthworms accompanied by a decrease in AChE and GST activities (Badawy et al. 2013). Al-Saeed et al. (2023) showed that prolonged exposure to lufenuron may have detrimental impacts on health by causing DNA damage, elevating oxidative stress, decreasing the profile of enzymatic antioxidants, and causing histological lesions in the visceral organs of Nile tilapita. Health of *Cyprinus carpio* was also negatively impacted by hepatic damage brought on by sublethal exposure to lufenuron due to oxidative stress (Ghelichpour et al. 2020).

To the best of our knowledge, the cytogenotoxic effects of lufenuron on *A. cepa* roots have not been previously documented. This study aims to evaluate the cytogenotoxic and DNA damaging effects of the insecticide lufenuron on *Allium cepa* root meristematic cells. To achieve this, we employed root growth inhibition, Allium anaphase-telophase, comet, RAPD-PCR and flow cytometry assays. We also performed in silico molecular docking analysis for offering a mechanistic viewpoint on the lufenuron’s cytogenotoxic effects.

## Materials and methods

### Chemicals

The insecticide Trowel^®^ 050 EC containing 50 g/L lufenuron was purchased from Hektaş (Turkey). Some properties of Lufenuron are given Table 1. All other chemicals were analytical grade and purchased from commercial suppliers.

**Table 1:** Some properties of Lufenuron

IUPAC Name	(RS)-1-[2,5-dichloro-4-(1,1,2,3,3,3-hexafluoropropoxy)phenyl]-3-(2,6-difluorobenzoyl)urea
**Synonyms**	fluphenacur; Lufenuron 97%;Lufenuron@1000 µg/mL in Acetone; N-((2,5-Dichloro-4-(1,1,2,3,3,3-hexafluoropropoxy)phenyl)carbaMoyl)-2,6-difluorobenzaMide; LUFENURON; Lufenurin; Lufenurone; cga-184,699;Lufenuronum; Lufenuron CRS
**Chemical structure**	
**Molecular Formula**	C_17_H_8_Cl_2_F_8_N_2_O_3_
**Molecular Weight**	511.15 g/mol
**CAS Number**	103055-07-8
**Water Solubility**	0.046 mg/L at 20 °C

### *A. cepa* root growth inhibition test and EC_50_ determination

Healthy, uniformly sized (2–3 cm in diameter) *A. cepa* bulbs (*2n = 16* chromosomes) were procured from a local market. The root growth inhibition assay and determination of the effective concentration causing 50% inhibition of root length (EC₅₀) for the insecticide lufenuron were conducted following the protocol established by Fiskesjö (1985), with modifications as suggested by Pirdal and Liman (2019). The commercial product was diluted in distilled water to obtain test concentrations (0.5, 1, 2, 3, 4, 5 and 6 mg/L) applied in this study. After removing the brown outer scales and trimming the dried roots, the bulbs were exposed to various concentrations of lufenuron for 96 h at room temperature in darkness. After exposure, ten roots per bulb (50 roots per concentration) were measured with a centimeter scale, and mean lengths were calculated to evaluate lufenuron’s phytotoxic effects. Lufenuron’s EC₅₀ value for *A. cepa* was found to be 2 mg/L based on a comparison of the treatment and control groups’ mean root length. All of the mentioned effects and EC_50_ values are with the commercial formulation Trowel^®^ 050 EC and not with analytical grade lufenuron.

### Allium anaphase-telophase test

The anaphase-telophase assay in *A. cepa* was conducted with modifications of previously reported procedures (Liman 2020; Rank and Nielsen 1994). Bulbs were germinated in distilled water under dark conditions at room temperature for 48 h until roots reached 1–2 cm. Roots were then treated with lufenuron at concentrations corresponding to 2×EC_50_ (4 mg/L), EC_50_ (2 mg/L), and ½×EC_50_ (1 mg/L), alongside a positive control (methyl methanesulfonate, 10 mg/L) and a negative control (distilled water), for 24, 48, 72, and 96 h in darkness. After exposure, roots were excised and fixed in Carnoy’s solution (ethanol: glacial acetic acid, 3:1 v/v) at 4 °C for 24 h, followed by replacement with 70% ethanol and storage at 4 °C. Hydrolysis was performed in 1 N HCl at 60 °C for 8–10 min, and root tips were stained with Schiff’s reagent for 25 min according to the squash technique (Ince Yardimci et al. 2022). For each treatment, ~ 1040–1140 cells were assessed for mitotic index (MI) and phase distribution, and other anomalies, and 100 anaphase–telophase cells were analyzed for chromosomal aberrations (CAs) using a Nikon Eclipse Ci-L microscope equipped with a CMOS camera (Argenit, Kameram5, Turkey). Five root tips per group were examined, and data were calculated using following equations.

$$\:MI\:\left(\%\right)=\frac{Total\:number\:of\:dividing\:cells}{Total\:number\:of\:cells}x100$$

$$\:Phase\:index\:\left(\%\right)=\frac{Particular\:phase\:}{Total\:number\:of\:dividing\:cells}x100$$

$$\:CA\:\left(\%\right)=\frac{Total\:aberrant\:cells}{100\:anaphase-telophase\:cells}x100$$

$$\:Other\:anomalies\:\left(\%\right)=\frac{Total\:other\:anomalies}{Number\:of\:total\:cells}x100$$

### Alkaline Comet Assay

**Fig. 1:**
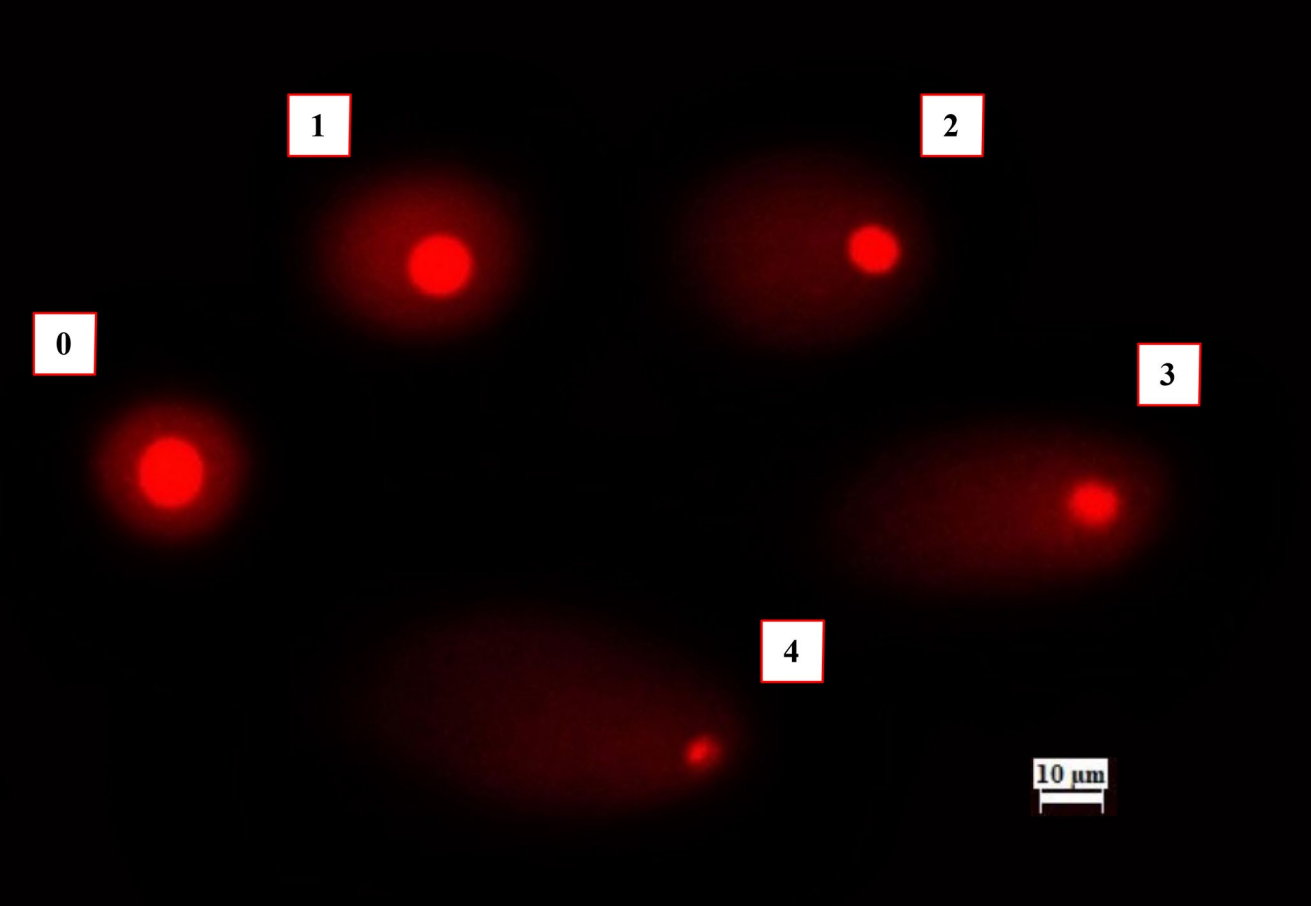
Lufenuron treated *A. cepa* root cells showed comet assay scores indicating DNA damage. Scores are as follows: 0 = < 5% (none), 1 = 5–20% (minor), 2 = 20–40% (moderate), 3 = 40–85% (severe), and 4 = > 85% (complete)

According to Tice et al. (2000), a modified comet assay was used to identify DNA damage in onion root cells induced by lufenuron. To encourage root growth, onions were grown in distilled water for two days at room temperature in the dark. After that, they were exposed to lufenuron for 24, 48, 72, and 96 h at doses of 1 mg/L, 2 mg/L, and 4 mg/L. The positive control was 10 mg/L MMS, whereas the negative control was distilled water. The root tips were finely chopped into 1-cm pieces using a scalpel, followed by the addition of 600 µL of ice-cold nuclei isolation buffer (4 mM MgCl₂·6 H₂O, 200 mM Tris, 0.5% Triton X-100). The onion root cell suspension was then filtered and centrifuged at 1200 rpm for 7 min. Slide preparation was performed according to the protocol described by Ince Yardimci et al. (2022). A 1:1 (v/v) mixture of 100 µL nuclear suspension and 1.5% low-melting-point agarose was spread onto a glass slide pre-coated with 1% normal-melting-point agarose and immediately covered with a coverslip. Slides were incubated in alkaline buffer (300 mM NaOH, 1 mM EDTA, pH > 13) at 4 °C for 20 min, followed by electrophoresis at 25 V, 300 mA, for 20 min at 4 °C. A fluorescence microscope (TAM-F, Turkey) was used to score 150 comets per sample (50 per slide) following treatment with ethidium bromide (20 µg/mL). As shown Fig. 1, DNA damage was classified into five categories (0–4) representing no (< 5%), minor (5–20%), moderate (20–40%), severe (40–85%), and complete damage (> 85%) by visual scoring and quantified using the standard arbitrary unit formula.

Arbitrary unit= $${\sum}_{i=0}^{4}\:{Nixi}$$

where Ni is the number of cells and i is the score (0, 1, 2, 3, 4) that indicates the extent of DNA damage.

### SubG1 analyses by Flow Cytometry

Three different concentrations (2×EC_50_, EC_50_, and ½×EC_50_) of the lufenuron, along with distilled water (negative control) and 10 mg/L MMS (positive control), were applied for 24 and 96 h to the root tips of *A. cepa* bulbs, which had been germinated in distilled water for 2 days. Flow cytometry analyses were conducted using three replicates for each concentration. Nuclear suspensions were prepared with slight modifications to the method described by Galbraith et al. (1983). Meristematic root cells of *A. cepa* were chopped with a sterile razor blade in a Petri dish containing 600 µL of nuclear isolation buffer, placed on ice. The nuclear suspensions were then filtered through a 45 μm nylon mesh to remove debris. Subsequently, 5 µL of RNase was added to each sample to eliminate RNA contamination. For analysis with the BD Accuri C6 Plus flow cytometer, the suspensions were stained with 50 µL of propidium iodide (1 mg/L), and SubG1 analyses were performed for determining the percentage of apoptotic cells in *A. cepa* root tip cells.

### Detection of Genomic Damage and Instability by RAPD-PCR Analysis

The i-genomic plant DNA mini kit (iNtRON Biotechnology, Korea) was used to extract genomic DNA from *A. cepa* roots following a 24 and 96-hours treatment with negative and positive control groups (distilled water and MMs) and lufenuron (1, 2 and 4 mg/L). Spectrophotometric analysis was used for checking the isolated genomic DNA’s content and purity. Prior to usage, the DNA supplies were kept at -20 °C. TaKaRa Ex Premier™ DNA Polymerase Dye plus kit (Takara Bio, Shiga, Japan) and five primers [OPB-04 (GGACTGGAGT), OPB-09 (TGGGGGACTC), OPB-12 (CCTTGACGCA), OBC-04 (CCGCATCTAC) and OPC-05 (GATGACCGCC)] were used for PCR. The thermal cycle program was as follow: 94 °C for 4 min followed by 94 °C for 45 s, 33–36 °C for, 72 °C for 1 min and 72 °C 10 min. The obtained PCR products were analyzed by electrophoresis on 1.4% agarose gel at 100 V for 1 h using 100 bp DNA Ladder (100–3000 bp) and DNA bands were stained with ethidium bromide and visualized under UV light. Gel images were subsequently analyzed using Bio-Rad ChemiDoc™ with Image Lab™ software to identify and distinguish polymorphic and monomorphic bands. The genomic template stability (GTS%) for each primer was determined using the following formula after RAPD profiles were compared to the negative control and polymorphism and band alterations were assessed. In addition, RAPD data were analyzed using Popgene version 32 software, and Unweighted Pair Group Method with Arithmetic Mean (UPGM)A cluster analysis was performed to quantitatively evaluate genetic relationships among the treatment groups.

GTS (%) = (1 - a/n)× 100.

Where the average number of changes in DNA profiles is a, and the number of bands used in control DNA profiles is n.

### Molecular docking studies

#### Rationale of Target Selection in Molecular docking

Lufenuron’s potential target receptors in plants remain unknown. However, the high level of cytogenotoxicity observed in the *A. cepa* roots suggest that lufenuron can interact with biological macromolecules at both the metabolic and nucleic acid levels. In the preliminary phase of our study, target prediction analysis using SwissADME server (http://www.swissadme.ch/) showed that this insecticide may inhibit human cytochrome P450 enzymes CYP2C19 and CYP2C9. CYP81A12 and CYP81A21 are plant cytochrome P450 monooxygenases that have been experimentally shown to catalyze the metabolism of xenobiotic compounds, thereby fulfilling detoxification functions analogous to those carried out by human CYP2C9 and CYP2C19 (Dimaano and Iwakami 2021; Goldberg-Cavalleri et al. 2025; Iwakami et al. 2014). Therefore, in our molecular docking study, these two cytochrome P450-type enzymes were selected as the target receptors for lufenuron. Furthermore, to elucidate lufenuron’s potential DNA interaction pattern and binding affinity, to unravel the DNA damage observed in the comet assay at the molecular level-lufenuron-DNA docking studies were performed. A double-stranded B-DNA dodecamer’s high-resolution X-ray crystal structure (PDB ID: 8CE2, resolution: 1.24 Å) from the Nucleic Acid Knowledgebase (NAKB) was utilized for this purpose (Tito et al. 2023).

### Ligand/Target preparation

This study utilized molecular docking simulations to investigate the cytogenotoxic effects of lufenuron on *A. cepa* root tip cells at the molecular scale. The metabolic fate of lufenuron in *Solanum lycopersicum*, *Brassica oleracea* var. *capitata*, and *Gossypium hirsutum* was examined by foliar spraying of radiolabeled ([dichlorophenyl-14 C] and/or [difluorophenyl-14 C]) lufenuron on these plants. Only unchanged lufenuron was found—primarily on treated surfaces—with no significant translocation after spraying or stem injection. It has been reported that, weeks later, leaves absorbed some residue, yet extracts contained only lufenuron (FAO 2015). Therefore, only the intact structure of lufenuron (no metabolites) was used in molecular docking experiments against the chosen target receptor structures. Moreover, we also performed pKa predictions of lufenuron using the MolGpKa server (https://xundrug.cn/molgpka). Pan et al. (2021) observed that the two NH groups of lufenuron have pKa values of approximately 8.5 and 9.0, indicating that at near-neutral pH (around 7–7.4) they remain predominantly protonated. Therefore, the neutral (non-deprotonated) form of lufenuron was used in subsequent docking calculations. For the docking studies, the homology-modelled structures of *Echinochloa phyllopogon* enzymes CYP81A12 (UniProtKB: R4WPI5, https://alphafold.ebi.ac.uk/entry/R4WPI5) and CYP81A21 (UniProtKB: A0A024FBW3, https://alphafold.ebi.ac.uk/entry/A0A024FBW3) were selected as the target receptors for lufenuron. The neutral conformer of lufenuron (Compound CID: 71777) was obtained from the PubChem database (https://pubchem.ncbi.nlm.nih.gov/compound/71777) in .sdf format, while the positive control, methyl methanesulfonate (MMS, Compound CID: 4156), used in the *A. cepa* assay, was similarly acquired in .sdf format. The DNA receptor’s crystal structure was preprocessed in Discovery Studio Visualizer (Biovia 2016) by eliminating water molecules and non-interacting ions prior to the docking simulations commencing.

In contrast, the CYP81A12 and CYP81A21 enzymes, generated as homology models *via* the AlphaFold server, required no such pretreatment. Next, these cytochromes were ionized at pH 7.4 using the PROPKA module in Vega ZZ (version 3.2.2.21) (Pedretti et al. 2021) and saved in mol2 format. The ligands—lufenuron and the positive control methyl methanesulfonate (MMS)—were energetically minimized using Avogadro program with the MMFF94 force field and the conjugate gradient algorithm (convergence = 10^− 7^), then also saved as mol2 files (Hanwell et al. 2012). Finally, the two ligands and receptor structures (CYP81A12, CYP81A21, and the dsDNA dodecamer) were converted to pdbqt format using AutoDock Tools 1.5.6 prior to conducting the docking experiments (Morris et al. 2009).

### Molecular docking

In this work, lufenurone and MMS’s binding interactions with CYP81A12, CYP81A21, and the dsDNA dodecamer were investigated in this study by molecular docking using AutoDock Vina (v1.2.5) (Eberhardt et al. 2021). During the preparation phase, polar hydrogens were preserved in both ligands and receptors, while non-polar hydrogens were merged using AutoDock Tools 1.5.6. Additionally, Kollman charges were applied to the receptor molecules and Gasteiger charges to the ligands. Docking simulations were performed using the semi-flexible protocol: while all ligand rotatable bonds were chosen to free rotation, the receptor structures remained rigid. Specifically, grid boxes for CYP81A12 were set to 19 × 19 × 19 Å with center coordinates (x: 4.76 Å; y: -0.73 Å; z: -6.83 Å) and for CYP81A21 to 21 × 21 × 21 Å with center coordinates (x: 4.67 Å; y: -0.12 Å; z: -6.37 Å). For the DNA receptor, a grid box of 80 × 70 × 100 Å (centered at x: 15.07 Å; y: 21.00 Å; z: 8.58 Å) was used. These settings ensured that the ligands could thoroughly interact with both the entire surface of the DNA molecule and the binding pockets of the CYP81A enzymes. The active regions (ligand binding pockets) of the CYP81A enzymes were identified using the *ˈDefine and Edit Binding Site*ˈ protocol in DS Visualizer. According to the DS Visualizer, this protocol analyzes the molecular surface of the protein to automatically detect potential cavities based on criteria such as cavity volume and surface area. The largest cavity, which is presumed to be the primary ligand binding site, was selected for each enzyme. The corresponding coordinates (see above) were then recorded and used in subsequent docking simulations. Binding affinity values (Δ*G*= kcal/mol) obtained from the CYP81A12-MMS, CYP81A21-MMS, and DNA-MMS docking experiments were used as control values for comparison with the results of the CYP81A12-lufenuron, CYP81A21-lufenuron, and DNA-lufenuron dockings.

For the docking experiments, 20 runs were performed with an *exhaustiveness* parameter of 64 for both lufenuron and MMS across all target receptors. Following docking, the resulting lufenuron conformations were clustered by geometric similarity by AutoDock Vina 1.2.5 and subsequently ranked by binding affinity; the conformation with the most negative binding free energy (Δ*G* = kcal/mol) was selected as the top-ranked pose. The top-ranked docking poses for lufenuron against the two CYP81A enzymes and DNA were then qualitatively assessed and visualized using Discovery Studio Visualizer (Biovia 2016).

### Statistical analysis

Data were analyzed using SPSS v23 and expressed as mean ± standard deviation. Group differences were evaluated by one-way ANOVA followed by Duncan’s post hoc test, while correlations between variables were assessed using Pearson’s test with significance set at *p* ≤ 0.05 and *p* < 0.01, respectively. Clustering analysis of RAPD PCR data was performed with the PopGene version 32 Software program.

## Results and discussion

### Cytogenotoxicity of Lufenuron on Allium roots

**Fig. 2:**
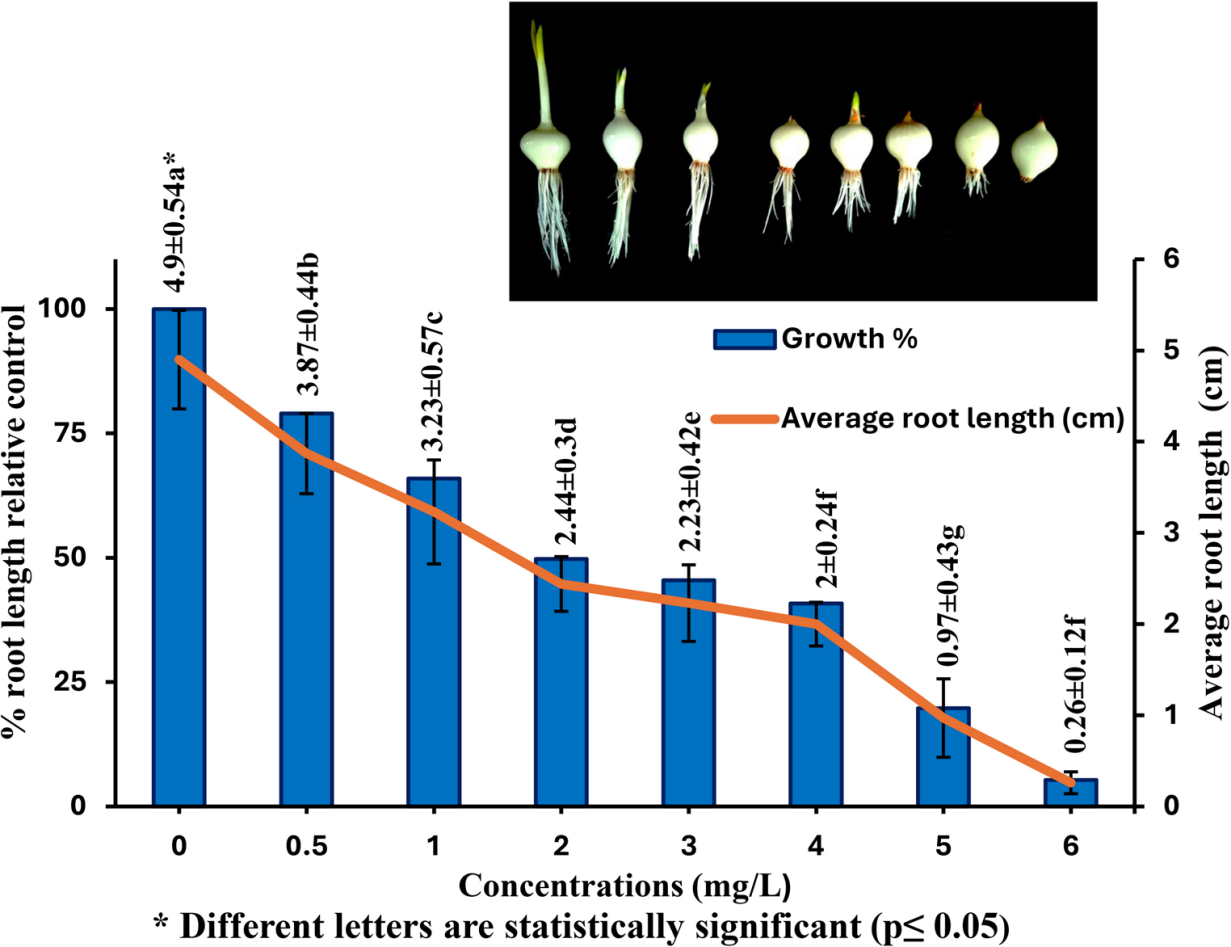
Root elongation in *A. cepa* after 96 h of exposure to control and lufenuron treatments

Lufenuron’s toxicity was assessed using the *A. cepa* root growth inhibition test (Fig. 2). Onion root lengths decreased significantly in a dose-dependent manner as lufenuron concentrations increased (*r*=-0.933; *p* < 0.001). Inhibition of root growth was noted in this way: 0.5 mg/L (3.87 ± 0.19 cm, 21.02%), 1 mg/L (3.23 ± 0.57 cm, 34.08%), 2 mg/L (2.44 ± 0.13 cm, 50.20%), 3 mg/L (2.23 ± 0.42 cm, 54.49%), 4 mg/L (2 ± 0.2 cm, 59.18%), 5 mg/L (0.97 ± 0.43 cm, 80.20%), and 6 mg/L (0.26 ± 0.12 cm, 94.69%). Lufenuron’s EC_50_ concentration was determined to be 2 mg/L. The shortening of the root length after lufenuron treatment could be explained by the inactivation of the enzymes involved in cell division and cell cycle regulation disruption (Fusconi et al. 2006; Silveira et al. 2017). Moreover, the inhibition of meristematic activity at the apex of the roots seems to be a key factor in the reported inhibition of root development (Macar 2021; Scherer et al. 2019). Yang et al. (2021) showed that the highly active chemical Y9k, 1-phenethyl-3- (3-(trifluoromethyl) phenyl) urea, exhibits activities linked to plant growth regulators and may successfully block Arabidopsis primary root elongation at 100 µmol/L (IC_50_ = 0.8 µmol/L).

The variations in the mitotic index (MI) and mitotic phase indices following the application of lufenuron to *A. cepa* roots are presented in Table 2. The treatment with lufenuron resulted in a statistically significant reduction in MI values compared to the control group, with a dose (*r* =- 0.934, -0.980, -0.980 and − 0.909 for 24, 48, 72, and 96 h, respectively; *p* < 0.001) and time dependent manner (*r* = -0.865, -0.859, and − 0.806 for 1, 2 and 4 mg/L, respectively; *p* < 0.001). Except for 72 h application, the reductions in MI at the 2 and 4 mg/L lufenuron dosages were even less pronounced than the MMS positive control group. High concentrations of lufenuron exposure statistically significantly reduced the prophase indices as compared to the control group, while all other indices—aside from the 24 h application at the anaphase index—rose significantly (*p* < 0.05). Prophase index lowering may be linked to both the antiproliferation effect of cell-cycle blocking in the G2-phase and a quicker prophase stage (Soliman and Ghoneam 2004). Lufenuron’s effect on the spindle and a delay in the mitotic cycle’s completion could be the cause of the rise in the metaphase and telophase indices, respectively (Singh and Roy 2017). de Souza et al. (2023) reported that anaphase–telophase arrest may indicate that lufenuron disrupts proteins essential for entry into the M phase. In this study, the decline in MI following lufenuron treatment appears to be linked to cell cycle inhibition, which could occur through impaired DNA synthesis or blockage of the G1–S and G2–M checkpoints (Aslantürk 2024; El-Ghamery et al. 2000; Sudhakar et al. 2001). Furthermore, reduced mitotic activity or changes in the duration of mitotic stages may result from exposure to toxic agents (Hidalgo et al. 1989; Saxena et al. 2005) or from anti-mitotic mechanisms involving the inhibition of DNA polymerase and other regulatory proteins controlling cell cycle progression (Hidalgo et al. 1989; Kaya et al. 2023; Turkoglu 2015).

**Table 2:** Effect of Lufenuron on mitotic and phase index in *A. cepa* roots

Concentration (mg/L)	CCN	MI ± SD*	Phase index (%) ± SD*			
			Prophase	Metaphase	Anaphase	Telophase
Control-24 h	5643	56.25 ± 0.9a	89.11 ± 1.12a	2.27 ± 0.31a	2.07 ± 0.36a	6.55 ± 0.76ac
MMS-10	5419	41.34 ± 0.9b	90.2 ± 0.91a	2.31 ± 0.57a	2.41 ± 0.43a	5.08 ± 0.57b
1	5238	50.07 ± 0.77c	90.12 ± 0.82a	2.09 ± 0.39a	1.99 ± 0.45a	5.8 ± 0.48ab
2	5435	39.99 ± 0.55d	90.04 ± 1.23a	2.34 ± 0.57a	1.97 ± 0.26a	5.65 ± 0.56b
4	5347	38.37 ± 0.86e	87.42 ± 0.37b	3.27 ± 0.25b	2.39 ± 0.44a	6.92 ± 0.63c
Control-48 h	5438	57.34 ± 0.79a	89.44 ± 0.79ac	2.69 ± 0.23a	2.08 ± 0.59a	5.79 ± 0.55a
MMS-10	5500	38.25 ± 0.32b	88 ± 0.91b	3.07 ± 0.55a	3.38 ± 0.73b	5.55 ± 0.24ab
1	5609	41.47 ± 1.04c	88.78 ± 0.76ab	3.06 ± 0.54a	2.99 ± 0.53b	5.17 ± 0.4b
2	5512	37.65 ± 1.06bd	90.12 ± 0.99c	2.56 ± 0.43a	2.95 ± 0.51b	4.37 ± 0.22c
4	5418	36.67 ± 0.6d	84.41 ± 0.97d	4.44 ± 0.78b	2.83 ± 0.33b	8.31 ± 0.6d
Control-72 h	5514	56.49 ± 1.23a	88.83 ± 0.82a	2.76 ± 0.34a	2.36 ± 0.49a	6.04 ± 0.47a
MMS-10	5664	29.95 ± 0.63b	91.51 ± 1.5b	2.01 ± 0.58b	1.64 ± 0.35b	4.85 ± 0.66b
1	5494	37.89 ± 0.48c	90.09 ± 1.07ab	2.8 ± 0.51a	2.07 ± 0.2ab	5.05 ± 0.81ab
2	5266	34.78 ± 0.57d	84.5 ± 0.98c	3.87 ± 0.68c	3.71 ± 0.61c	7.92 ± 0.69c
4	5292	29.97 ± 0.7b	84.1 ± 0.88c	4.2 ± 0.64c	4.12 ± 0.48c	7.58 ± 1.16c
Control-96 h	5337	56.99 ± 0.41a	90.24 ± 0.79a	2.5 ± 0.33a	1.97 ± 0.25a	5.29 ± 0.39a
MMS-10	5626	29.43 ± 0.3b	86.08 ± 0.96b	3.64 ± 0.68b	3.2 ± 0.53b	7.08 ± 0.48b
1	5312	31.54 ± 0.64c	90.76 ± 1.16a	3.1 ± 0.64ab	1.97 ± 0.38a	4.17 ± 0.51c
2	5295	28.82 ± 0.77b	80.42 ± 0.86c	3.8 ± 0.35b	4.12 ± 0.39c	11.66 ± 0.71d
4	5545	27.79 ± 0.51d	74.07 ± 1.99d	7.58 ± 1.12c	4.6 ± 0.87c	13.75 ± 0.96e

* Different letters in the same columns for each treatment time are statistically significant (*P* ≤ 0.05). CCN: Counting Cell Numbers, MI: Mitotic Index, SD: Standard Deviation

**Fig. 3:**
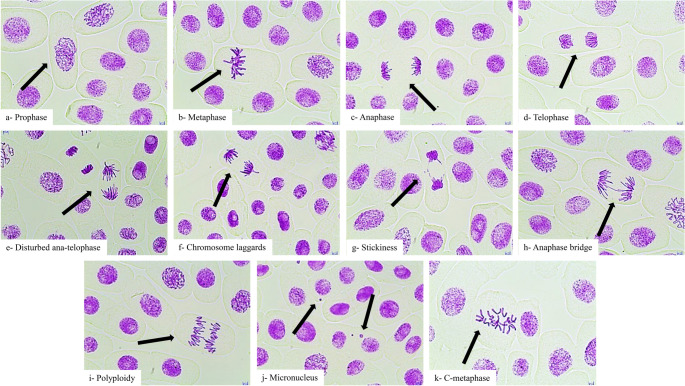
Microscopic images showing mitotic divisions and chromosomal aberrations in *A. cepa* root tips following lufenuron treatment. Scale bar: 10 μm, 400×

**Fig. 4:**
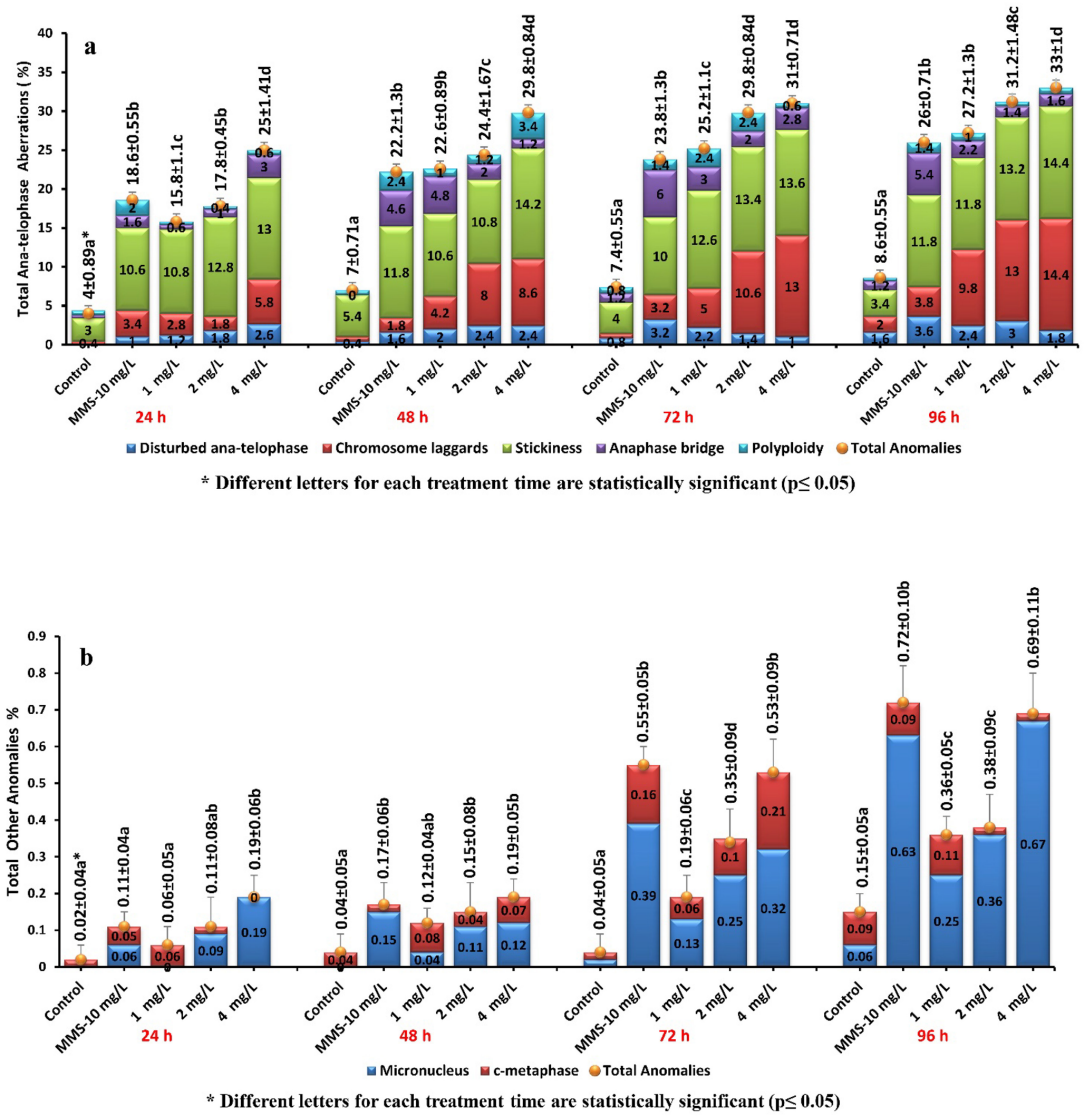
Frequency of CAs in *A. cepa* anaphase-telophase cells (a) and other cells (b) exposed to lufenuron at different concentrations and intervals

The root meristem of *A. cepa* treated with different concentrations of lufenuron showed different types and frequencies of CAs (Figs. 3 and 4), according to cytological examination by using Allium anaphase-telophase test. Total CAs in anaphase–telophase cells increased progressively with rising lufenuron concentrations (*r* = 0.924, 0.907, 0.902, and 0.884 at 24, 48, 72, and 96 h, respectively; *p* < 0.001) and with prolonged exposure duration (*r* = 0.931, 0.947, and 0.914 at 1, 2, and 4 mg/L, respectively; *p* < 0.001). Lufenuron treatment primarily induced stickiness, followed by laggards, anaphase bridges, disturbed anaphase-telophase, and polyploidy in anaphase-telophase cells. Sticky chromosomes (Fig. 3g), a form of chromatid aberration, may arise from DNA depolymerization, improper condensation, fragmentation, or breakage, and can contribute to chromosomal bridge formation (Barman and Ray 2023; Fatma et al. 2018; Haq et al. 2016). Chromosomal laggards (Fig. 3f) and disturbed anaphase-telophase (Fig. 3e) likely result from spindle fiber defects that impair the transport of chromosomes to the poles (Das and Ray 2024; Kizilkaya et al. 2023). Anaphase bridges (Fig. 3h) may form due to chromosomal breaks, dicentrics, faulty chromatid exchanges, replication disturbances, or clastogenic activity of insecticides (El-Ghamery et al., 2000; Beatriz Andrioli et al. 2022; Das and Ray 2024). Additionally, polyploidy (Fig. 3i) may develop when chromosomes fail to separate correctly during mitosis (Cildir and Liman 2020). Apart from anaphase-telophase abnormalities, micronuclei and c-metaphase formation were also detected in other cells depending on the concentration (Fig. 4b). Micronuclei (Fig. 3j) are DNA fragments that can form due to defects in spindle attachment or through various pathways, including the generation of acentric chromosome fragments resulting from unrepaired double-stranded DNA breaks. Additionally, they may arise from the simultaneous excision repair of damaged or improperly incorporated bases in DNA when these lesions occur in close proximity on complementary DNA strands (Fenech et al. 2011; Kassa 2021). c-metaphase (Fig. 3k) may develop when spindle apparatus function is disrupted or when chromatin-associated proteins are imbalanced, affecting nuclear organization (Mesi and Kopliku 2013). Chromosome aberrations in *Drosophila melanogaster* (Awad et al. 2018), and micronuclei formation in the erythrocytes of male Japanese quails (Saeed et al. 2025) were also recorded after exposure to lufenuron. The cytogenotoxic effects obtained for the formulated lufenuron product are broadly consistent with reports for other benzoylurea insecticides. Bıyıksız et al. (2025) showed that triflumuron induced multifaceted toxicity by limiting germination and growth, inducing cytogenotoxic effects, disrupting oxidative balance, and altering the structure of meristematic cells in *A. cepa*.

**Fig. 5:**
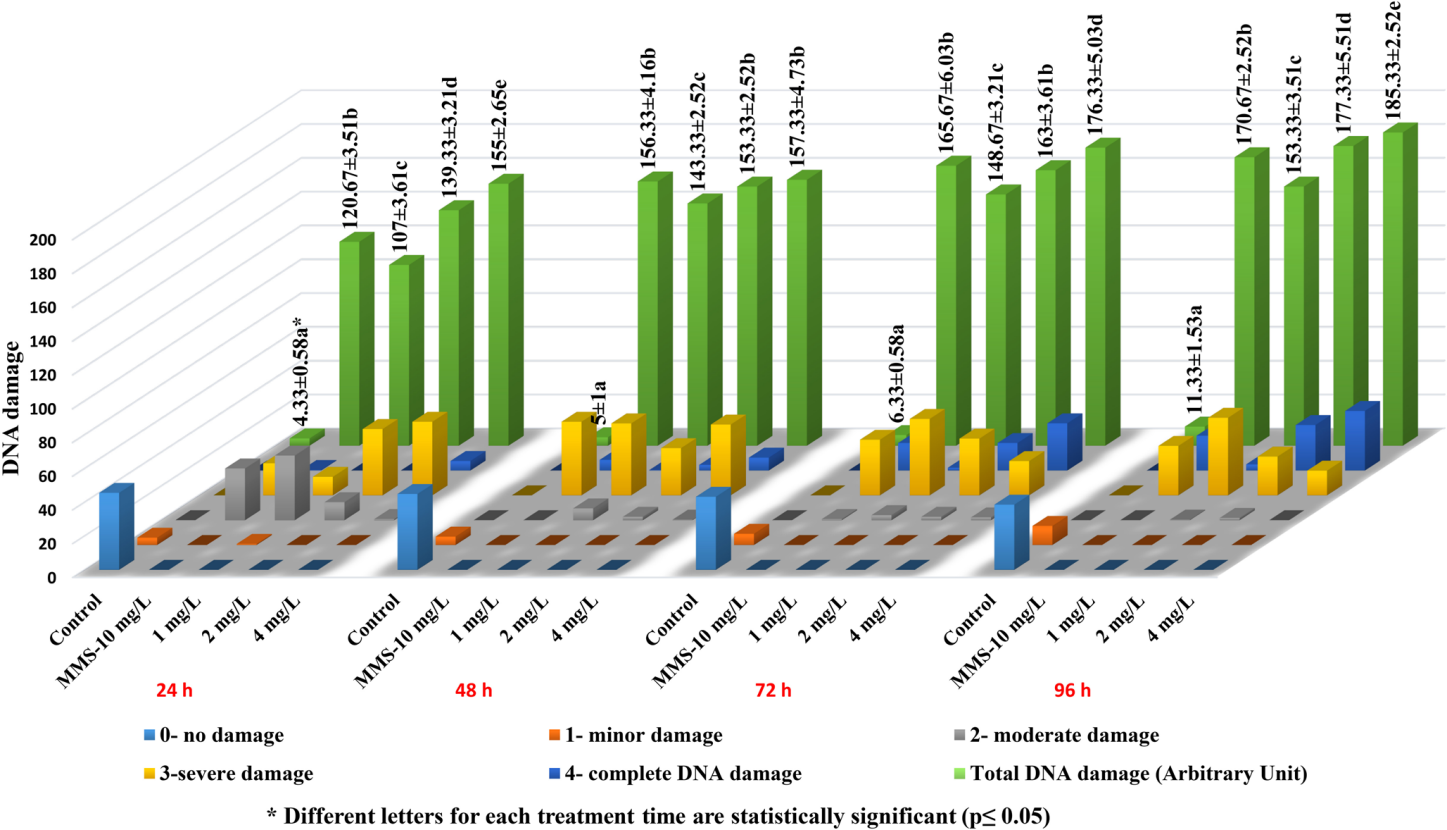
Lufenuron-induced DNA damage in *A. cepa* roots at different concentrations and intervals

Comet assay analysis demonstrated that lufenuron caused a significant increase in DNA damage in *A. cepa* root tip cells at all exposure times (Fig. 5). DNA damage induced by lufenuron showed strong positive correlations across concentrations at all exposure times (*r* = 0.972 at 24 h; *p* < 0.001, 0.877 at 48 h; *p* = 0.002, 0.960 at 72 h; *p* < 0.001, and 0.934 at 96 h; *p* < 0.001). This trend persisted across exposure durations for each tested concentration (*r* = 0.873 at 1 mg/L, 0.972 at 2 mg/L, and 0.935 at 4 mg/L; *p* < 0.001). At 4 mg/L, DNA damage exceeded that of the positive control (MMS), with significance detected at all points in time except 48 h. To date, no studies have directly investigated lufenuron-induced DNA damage in plants using the comet assay. However, existing research shows that lufenuron may trigger oxidative stress through increased ROS production, free radical generation, and enzyme activity disruption in non-target organisms, including pregnant albino rats and their fetuses (Basal et al. 2020, 2021), *Oreochromis niloticus* (Al-Saeed et al. 2023), male Japanese quail (Saeed et al. 2025), and *Biomphalaria alexandrina* snails (Ibrahim et al. 2018). These findings underscore the broad toxicological relevance of lufenuron across diverse biological systems; nevertheless, considering the fundamental differences in cellular architecture and stress-response pathways, any resemblance to plant-related mechanisms should be approached with caution and regarded as indicative rather than directly demonstrative.

### SubG1 Analysis

**Fig. 6:**
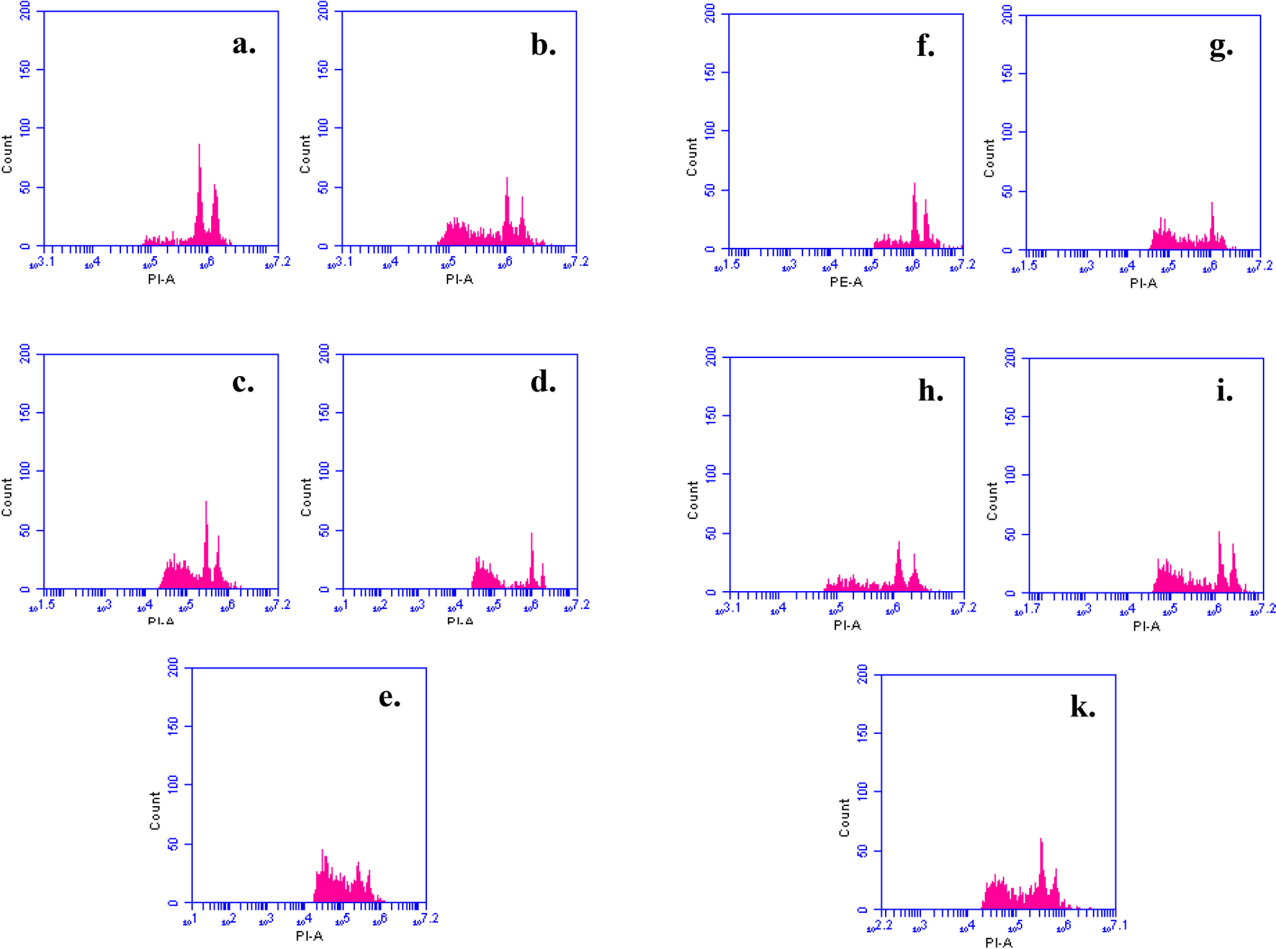
Sub-G1 phase analysis of *A. cepa* root meristem cells after 24-hours (a-e) and 96- hours (f-k) exposure to lufenuron. Flow-cytometry histograms (propidium iodide staining) show: (a and f) negative controls, (b and g) positive controls (MMS 10 mg/L), (c and h) Lufenuron 1 mg/L, (d and i) Lufenuron 2 mg/L, and (e and k) Lufenuron 4 mg /L

Cells that have experienced DNA loss in the latter phases of apoptosis can be identified using the subG1 peak analysis approach. According to the subG1 analysis, lufenuron caused a statistically significant increase in apoptosis in *A. cepa* root tip cells across all exposure times (Fig. 6; Table 3). Strong positive correlations were observed at both sampling times (*r* = 0.972 24 h and *r* = 0.971 for 96 h; *p* < 0.001), and this trend persisted across concentrations, with correlation coefficients of *r* = 0.983 *p* < 0.001, 0.922 *p* = 0.009, and 0.971 *p* = 0.001 at 1, 2, and 4 mg/L, respectively. Only 40.53% and 55.23% of the cells in the positive control group (MMS) were detected in the subG1 peak region, whereas 55.43% and 63.37% of cells at a 4 mg/L concentration of lufenuron were found in this region for 24 and 96 h, respectively. Cell cycle perturbations after lufenuron exposure were also detected in liver cells of pregnant albino rats and their fetuses (Basal et al. 2020, 2021) and in liver of Atlantic salmon (Olsvik et al. 2024).

**Table 3:** The percentages of cells undergoing apoptosis represented by subG1 in *A. cepa* root tip cells induced by lufenuron,

Treatment	Concentration (mg/L)	Percentage of subG1 peak Mean ± Standard Deviation*	
		24 h	96 h
**Control**	**-**	19,60 ± 1,35a	23,57 ± 1,35a
**MMS**	**10**	40,53 ± 0,47b	55,23 ± 2,5b
**Lufenuron**	**1**	36,37 ± 0,83c	49,87 ± 2,05c
	**2**	47,67 ± 2,93d	56,87 ± 1,6b
	**4**	55,43 ± 1,90e	68,37 ± 1,97d

* Different letters in the same columns for each treatment time are statistically significant (*P* ≤ 0.05).

### RAPD-PCR analysis of genomic template stability and DNA polymorphism

**Fig. 7:**
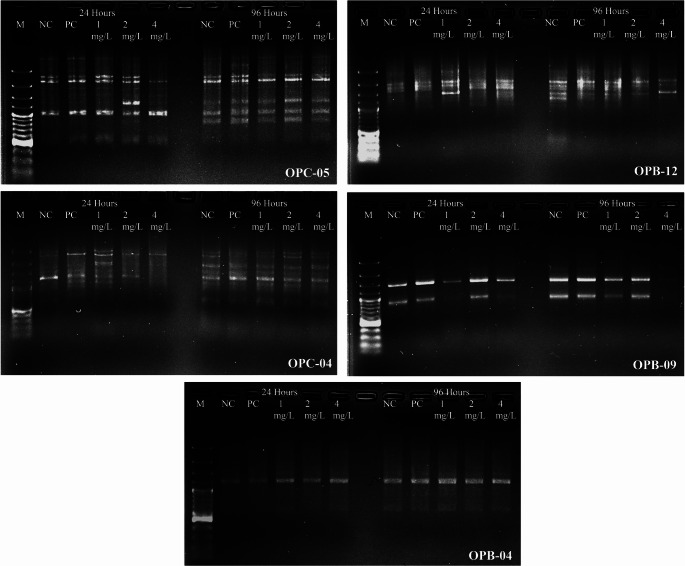
RAPD profiles in *A. cepa* L. root meristem cells exposed to untreated control (NC), positive control (PC: MMS-10 mg/L), and lufenuron treatments at EC_50/2_, EC_50_ and EC_50 × 2_ concentrations for 24 and 96 h. RAPD profiles were generated using primers OPB-04, OPB-09, OPB-12, OBC-04 and OPC-05. M: GeneRuler 100 bp Plus DNA Ladder (100–3000 bp)

**Fig. 8:**
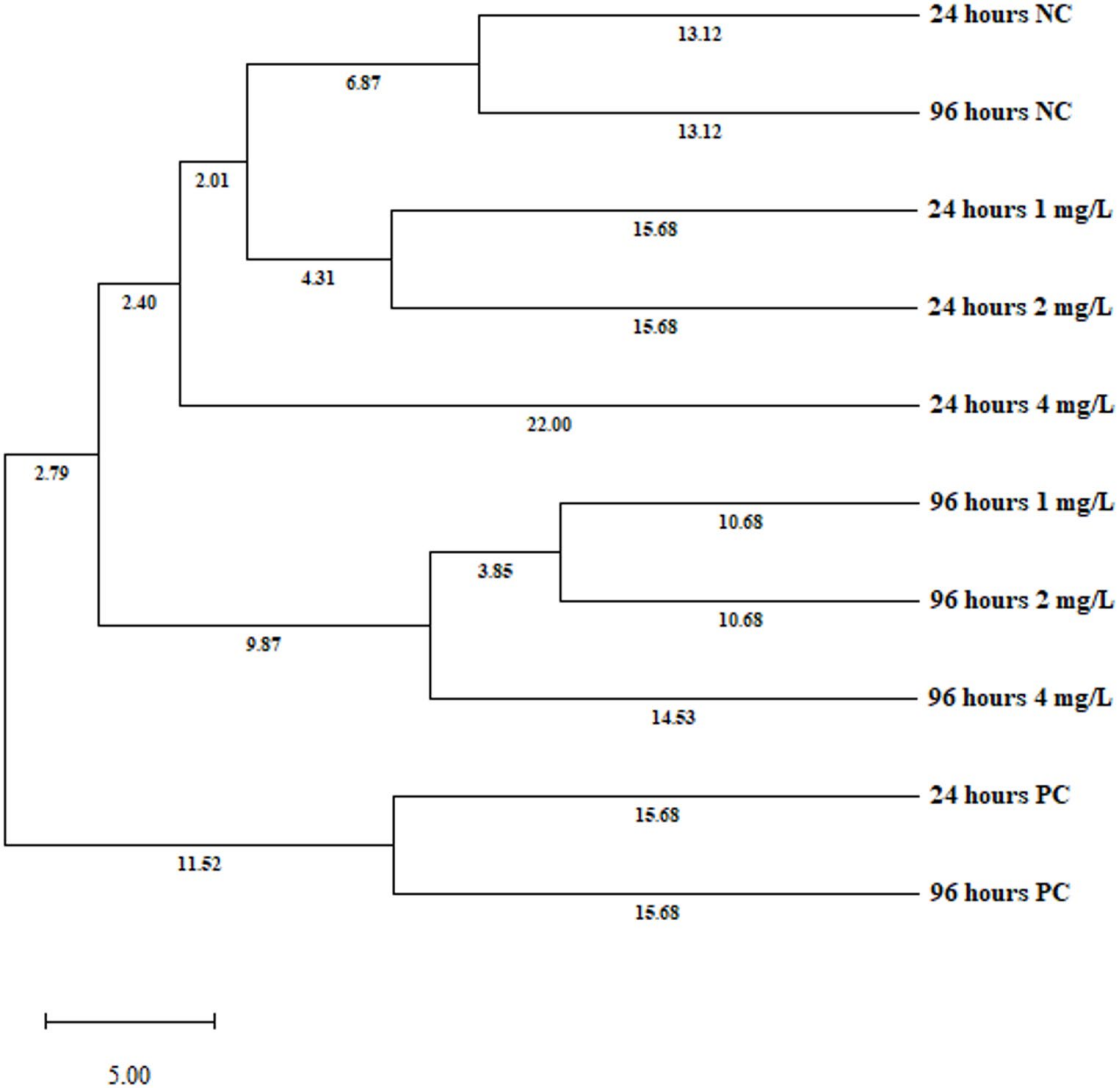
Dendrogram using UPGMA of DNA polymorphism in *A. cepa* L. root meristem cells treated with lufenuron at different concentrations and exposure times. NC: Negative control PC: Positive control (MMS-10 mg/L)

RAPD-PCR analysis was used as a semi-quantitative genomic screening approach to evaluate DNA instability through band pattern variations and genomic template stability indices. RAPD profiles generated with five primers (OPB-04, OPB-09, OPB-12, OBC-04, and OPC-05) revealed significant genomic alterations in *A. cepa* root tip cells following lufenuron exposure (Fig. 7; Tables 4 and 5). While OPB-04 produced a single monomorphic band in both treated and control groups, the other primers displayed clear band losses and the appearance of novel fragments, confirming DNA template instability. Particularly, OPB-09 and OPB-12 primers showed complete band loss at 24 h, indicating a strong reduction in GTS (Table 4). According to total band analysis, the monomorphism rate was 7.27% and the polymorphism rate was 52.73% at 24 h. At 96 h, the monomorphism rate increased slightly to 8.33% while the polymorphism rate rose to 58.33% (Table 5). These results demonstrate that DNA variability increases with exposure duration. Notably, primers OBC-04 and OPB-09 generated the highest numbers of polymorphic bands, highlighting the pronounced genotoxic effect of lufenuron on genetic diversity. In this context, the reduction in GTS values represents a quantitative measure of accumulated DNA alterations, as each band loss or gain reflects structural changes within the genome induced by genotoxic stress. Therefore, GTS (%) serves not only as a descriptive parameter but also as a numerical indicator of genomic instability. The dendrogram generated by UPGMA clustering based on Popgene32 analysis (Fig. 8) not only illustrated the grouping pattern but also highlighted the genetic distances among treatments. The control and low-dose groups (1 and 2 mg/L, 24 h) clustered tightly with minimal branch lengths, indicating high genomic similarity and stability under mild exposure. In contrast, high-dose treatments (4 mg/L at 24 and 96 h) showed markedly longer branch lengths from both controls and low-dose groups, reflecting substantial genomic divergence. Negative controls from both exposure times consistently formed the closest cluster, whereas positive controls were positioned at a distant branch with the largest genetic distances, confirming their pronounced genotoxic effect. Furthermore, the intermediate branch distances observed in the 96 h low-dose treatments suggest that prolonged exposure, even at lower concentrations, gradually accumulates genomic alterations. Importantly, the genetic distance matrix underlying UPGMA clustering is derived from cumulative RAPD band polymorphisms and thus provides a quantitative representation of DNA damage severity across treatments. The progressive increase in genetic distance values parallels the dose- and time-dependent increases observed in comet assay DNA damage scores and CAs frequencies, supporting the direct association between the RAPD-derived matrix and genotoxicity.

**Table 4:** Genomic template stability (GTS, %) in *A. cepa* L. root meristem cells exposed to different concentrations of Lufenuron insecticide for 24 and 96 h. Negative control (distilled water) and positive control (MMS, 10 mg/L) are included

	24 h					96 h				
	NC	PC	1 mg/L	2 mg/L	4 mg/L	NC	PC	1 mg/L	2 mg/L	4 mg/L
**OPC-05**	100,0	37.5	37.5	37.5	0	100,0	42.9	42.9	42.9	42.9
**OPC-04**	100.0	0.0	0.0	0.0	0.0	100,0	0.0	12.5	0.0	0.0
**OPB-12**	100.0	0.0	0.0	0.0	28.6	100,0	14.3	28.6	0.0	0.0
**OPB-09**	100.0	0.0	0.0	0.0	0.0	100.0	0.0	0.0	0.0	0.0
**OPB-04**	100.0	100.0	100.0	100.0	100.0	100.0	100.0	100.0	100.0	100.0

**Table 5:** Primers used in RAPD-PCR analysis, molecular sizes of DNA bands, control band numbers, and counts of monomorphic and polymorphic bands obtained from *A. cepa* L. root meristem cells exposed to Lufenuron for 24 and 96 h

Hours	Primers	G + C (%)	5’>3’ Sequence	Largest DNA Band (bp)	Smallest DNA Band (bp)	Monomorphic Band Number	Polymorphic Band Number		Total Band Number
**24 h**	OPC-05	70.00	GATGACCGCC	4000	625	3	4		11
	OBC-04	60.00	CCGCATCTAC	3000	738	0	7		18
	OPB-12	60.00	CCTTGACGCA	2920	1268	0	6		13
	OPB-04	60.00	GGACTGGAGT	1279	1279	1	0		1
	OPB-09	70.00	TGGGGGACTC	2471	1047	0	12		12
					**Total**	4	29		55
						**Monomorphism %**		7.27	
						**Polymorphism %**		52.73	
**96 h**	OPC-05	70.00	GATGACCGCC	4000	941	**2**		4	11
	OBC-04	60.00	CCGCATCTAC	2980	698	**1**		7	16
	OPB-12	60.00	CCTTGACGCA	2775	1342	**0**		7	10
	OPB-04	60.00	GGACTGGAGT	1279	1279	**1**		0	1
	OPB-09	70.00	TGGGGGACTC	2490	931	**0**		10	10
					**Total**	**4**		28	48
						**Monomorphism %**		8.33	
						**Polymorphism %**		58.33	

These findings further confirm that lufenuron induces pronounced genomic template instability in *A. cepa*. Comparable RAPD alterations, such as band disappearance, the emergence of novel fragments, and reductions in GTS values, were also reported by (Shaaban et al., 2007) in *Spodoptera littoralis* strains treated with lufenuron, where extensive DNA polymorphism was observed as an indicator of strong genotoxic stress. Likewise, Mahmoud and Kamel (2019) demonstrated that another benzoylurea insecticide, teflubenzuron, triggered similar RAPD-PCR alterations in *Tribolium castaneum*, including the loss and appearance of bands that reflect diminished genomic template stability. Taken together, these studies indicate that benzoylurea compounds, including lufenuron and teflubenzuron, exert consistent genotoxic influences detectable by RAPD markers. The recurrence of comparable DNA polymorphism patterns across different biological systems highlights genomic instability as a characteristic response to benzoylurea exposure and underscores the sensitivity of RAPD-PCR in detecting such alterations.

### Lufenuron–CYP81A12 interactions

**Fig. 9:**
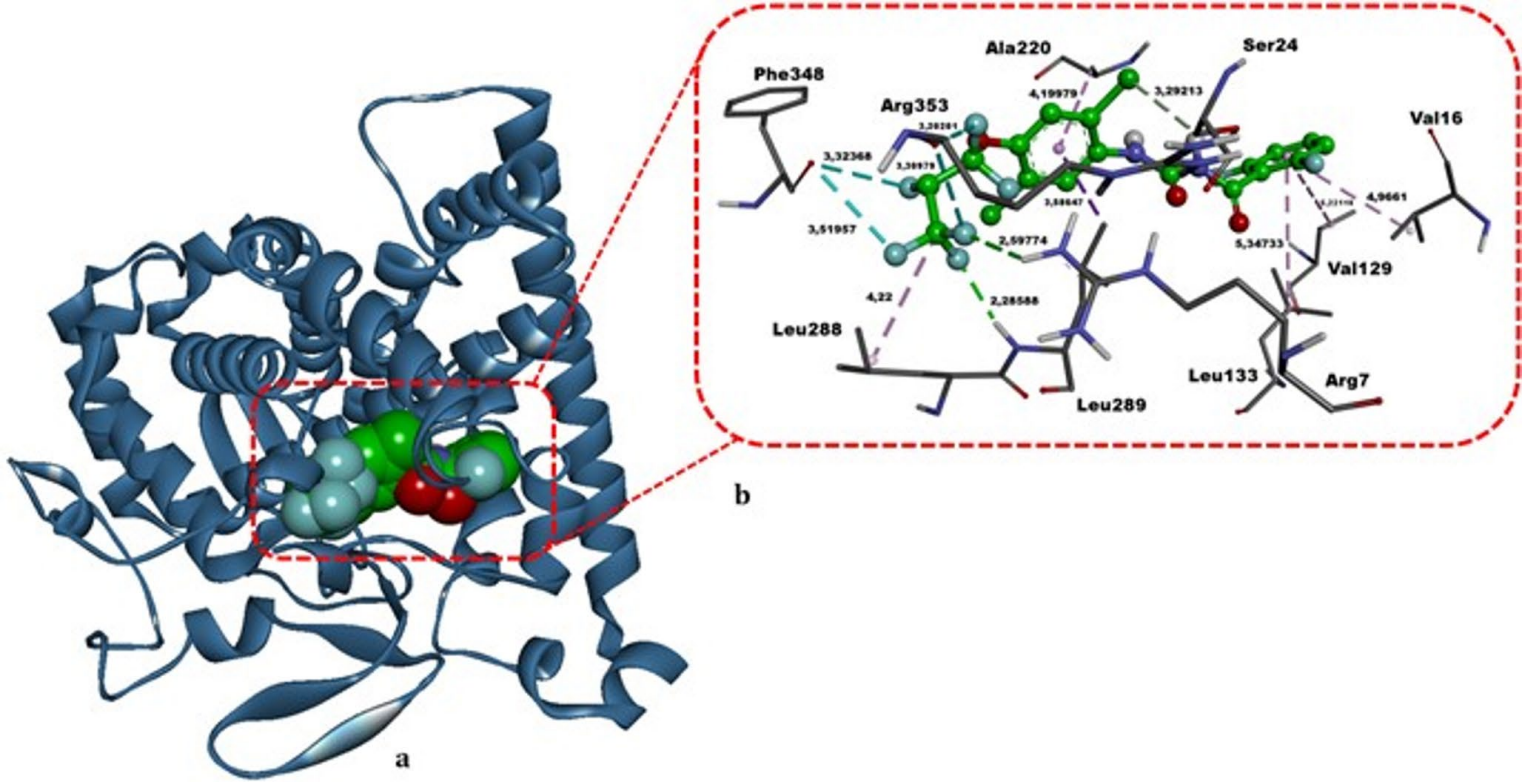
The top-scoring binding conformation of lufenuron complexed with the *Echinochloa phyllopogon* CYP81A12 enzyme. Panel **a** highlights a three‐dimensional overview of the CYP81A12–lufenuron complex, while Panel **b** shows a detailed view of lufenuron’s positioning within the enzyme’s active site. Hydrogen bonds are depicted by green and light green dashed lines, hydrophobic interactions by dark and light purple dashed lines, and halogen contacts by light cyan dashed lines. Bold black annotations indicate the non‐bonded interaction distances (in Å) and residue names. All structural modeling and visualization were carried out using DS Studio v16

The molecular docking of lufenuron within the CYP81A12 active site (Table 6; Fig. 9) revealed a binding free energy of − 9.78 kcal/mol, markedly stronger than that of the positive control MMS (ΔG= − 3.58 kcal/mol). Lufenuron forms classical hydrogen bonds with Arg7 (2.60 Å) and Leu289 (2.29 Å) as well as a carbon–hydrogen bond with Ser24 (3.29 Å). Moreover, it engages in several hydrophobic contacts involving Val16 (4.97 Å), Val129 (5.22 Å), Leu133 (5.35 Å), Ala220 (4.20 Å), Leu288 (4.22 Å), and Leu289 (3.59 Å), alongside halogen interactions with Phe348 (3.32 Å, 3.52 Å) and Arg353 (3.20 Å, 3.31 Å). These multifaceted interactions underscore lufenuron’s robust binding affinity toward CYP81A12. Cytochrome P450 enzymes belonging to the CYP81A subfamily are critically involved in the metabolic detoxification of xenobiotics in plant cells. Therefore, strong binding of lufenuron to the CYP81A12 active site may compromise enzymatic detoxification capacity, potentially facilitating intracellular persistence and accumulation of the compound, which in turn may contribute to the genotoxic outcomes observed in *A. cepa* root meristematic cells.

**Table 6:** Docking binding free energies (kcal/mol) and CYP81A-interaction results for Lufenuron and positive control Methyl methanesulfonate (MMS)

Compound							Hydrophobic interaction							
	Molecular weight (g/mol)	Receptor	ΔG_best_ (kcal/mol)	Classical H-bond		Non-classical H-bond (carbon-hydrogen)		Alkyl/ π-alkyl, π-sigma interaction		Halogen (fluorine)			Misc. (pi-sulfur)	
MMS (positive control)	110.13	CYP81A12*	-3.58	Arg7 (2.21 Å, 3.00 Å), Ser24 (3.34 Å), Arg36 (2.78 Å), Arg353 (2.45 Å, 2.56 Å, 3.24 Å)		—		—		—			Trp32 (5.63 Å)	
MMS (positive control)	110.13	CYP81A21**	-3.68	Arg7 (2.09 Å, 2.99 Å), Ser24 (3.23 Å), Arg36 (2.62 Å), Arg356 (2.38 Å, 2.45 Å, 3.23 Å)		—		—		—			Trp32 (5.63 Å)	
Lufenuron	470.31	CYP81A12	-9.78	*Arg7* (2.60 Å), Leu289 (2.29 Å)		*Ser24* (3.29 Å)		Val16 (4.97 Å), Val129 (5.22 Å), Leu133 (5.35 Å), Ala220 (4.20 Å), Leu288 (4.22 Å), Leu289 (3.59 Å)		Phe348 (3.32 Å, 3.52 Å), *Arg353* (3.20 Å, 3.31 Å)			—	
Lufenuron	470.31	CYP81A21	-10.00	Arg165 (2.86 Å)		*Ser24* (3.75 Å), Arg165 (3.18 Å, 3.33 Å), Gly220 (3.68 Å)		Ile125 (5.48 Å), Ile126 (5.11 Å), Val129 (3.85 Å), Val130 (4.13 Å), Arg165 (4.66 Å), Ala221 (5.00 Å), Ala286 (4.69 Å), Cys358 (4.66 Å), Leu397 (4.43 Å)		Ile125 (3.43 Å), Ala221 (3.65 Å)			—	

* (UniProtKB ID: R4WPI5): Cytochrome P450, CYP81A12, *Echinochloa phyllopogon*

**(UniProtKB ID: A0A024FBW3): Cytochrome P450, CYP81A21, *Echinochloa phyllopogon*

Δ*G*_best_: Binding free energy (*kcal/mol*) of the most favorable pose.

The underlined residues (Arg7, Ser24, and Arg353) indicate that lufenurone is predicted to engage the same binding pocket as the experimental positive control in our docking simulations. This concurrence supports the robustness of the docking search algorithm employed.

### Lufenuron–CYP81A21 interactions

**Fig. 10:**
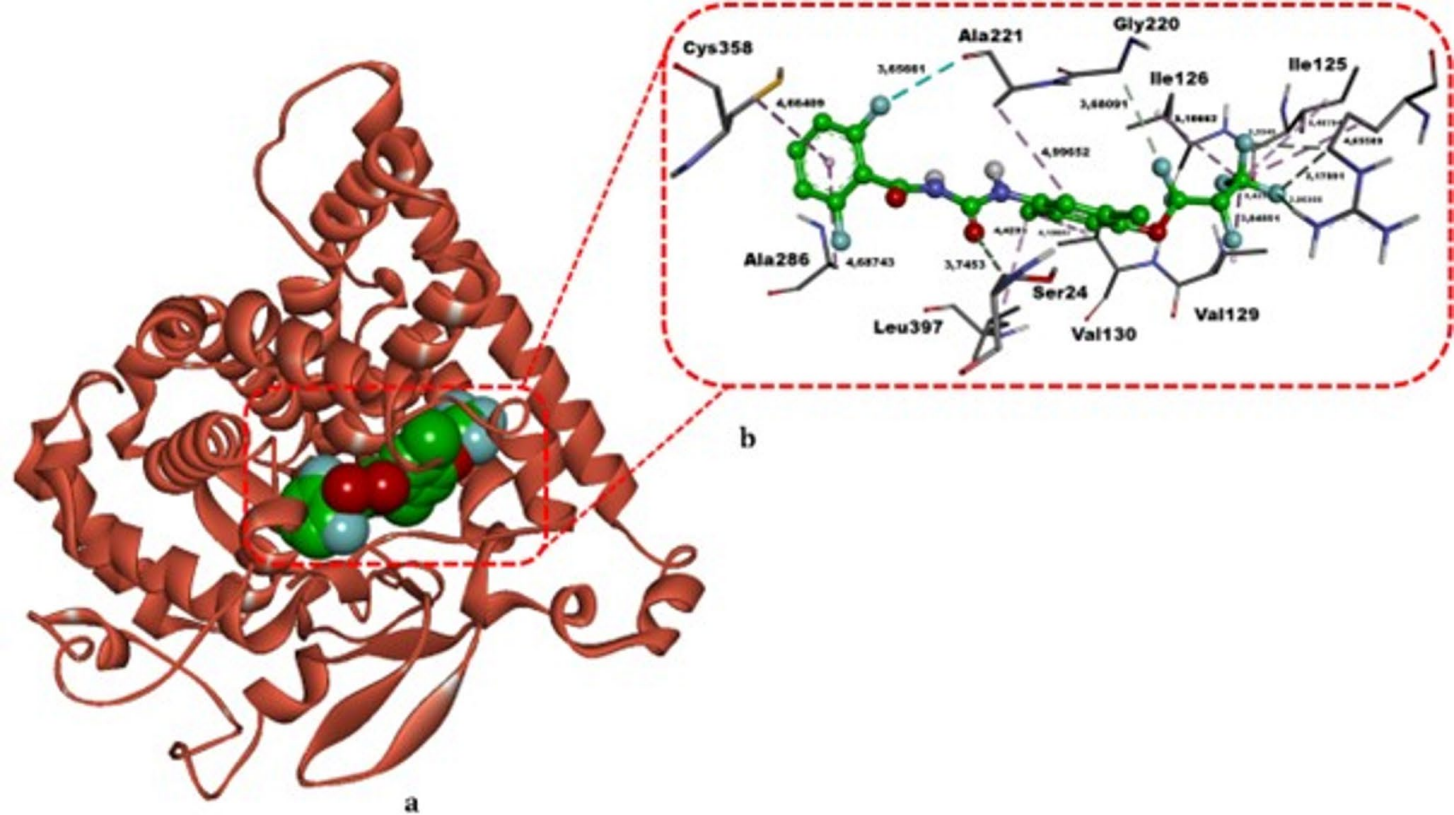
The top-scoring binding conformation of lufenuron complexed with the *Echinochloa phyllopogon* CYP81A21 enzyme. Panel **a** highlights a three‐dimensional overview of the CYP81A21–lufenuron complex, while Panel **b** shows a detailed view of lufenuron’s positioning within the enzyme’s active site. Hydrogen bonds are depicted by green and light green dashed lines, hydrophobic interactions by light purple dashed lines, and halogen contacts by cyan dashed lines. Bold black annotations indicate the non‐bonded interaction distances (in Å) and residue names. All structural modeling and visualization were carried out using DS Studio v16

The molecular docking of lufenuron in the active site of CYP81A21 (Table 6; Fig. 10) revealed a binding free energy of − 10.00 kcal/mol, substantially stronger than that of the positive control MMS (ΔG = − 3.68 kcal/mol). Lufenuron forms one classical hydrogen bond with Arg165 (2.86 Å) and establishes non-classical carbon–hydrogen bonds with Ser24 (3.75 Å), Arg165 (3.18 Å and 3.33 Å), and Gly220 (3.68 Å). Additionally, it engages in hydrophobic interactions with Ile125 (5.48 Å), Ile126 (5.11 Å), Val129 (3.85 Å), Val130 (4.13 Å), Arg165 (4.66 Å), Ala221 (5.00 Å), Ala286 (4.69 Å), Cys358 (4.66 Å), and Leu397 (4.43 Å), as well as halogen (fluorine) interactions with Ile125 (3.43 Å) and Ala221 (3.65 Å). Collectively, the range of interactions confirms lufenuron’s potent affinity against CYP81A21. Given the established role of CYP81A21 in plant xenobiotic metabolism, its pronounced interaction with lufenuron suggests a potential inhibitory effect on detoxification pathways. Such inhibition may prolong cellular exposure to unmetabolized lufenuron, thereby increasing the likelihood of secondary cellular stress responses and accumulation-associated genotoxic effects.

### Lufenuron—DNA Interactions

**Fig. 11:**
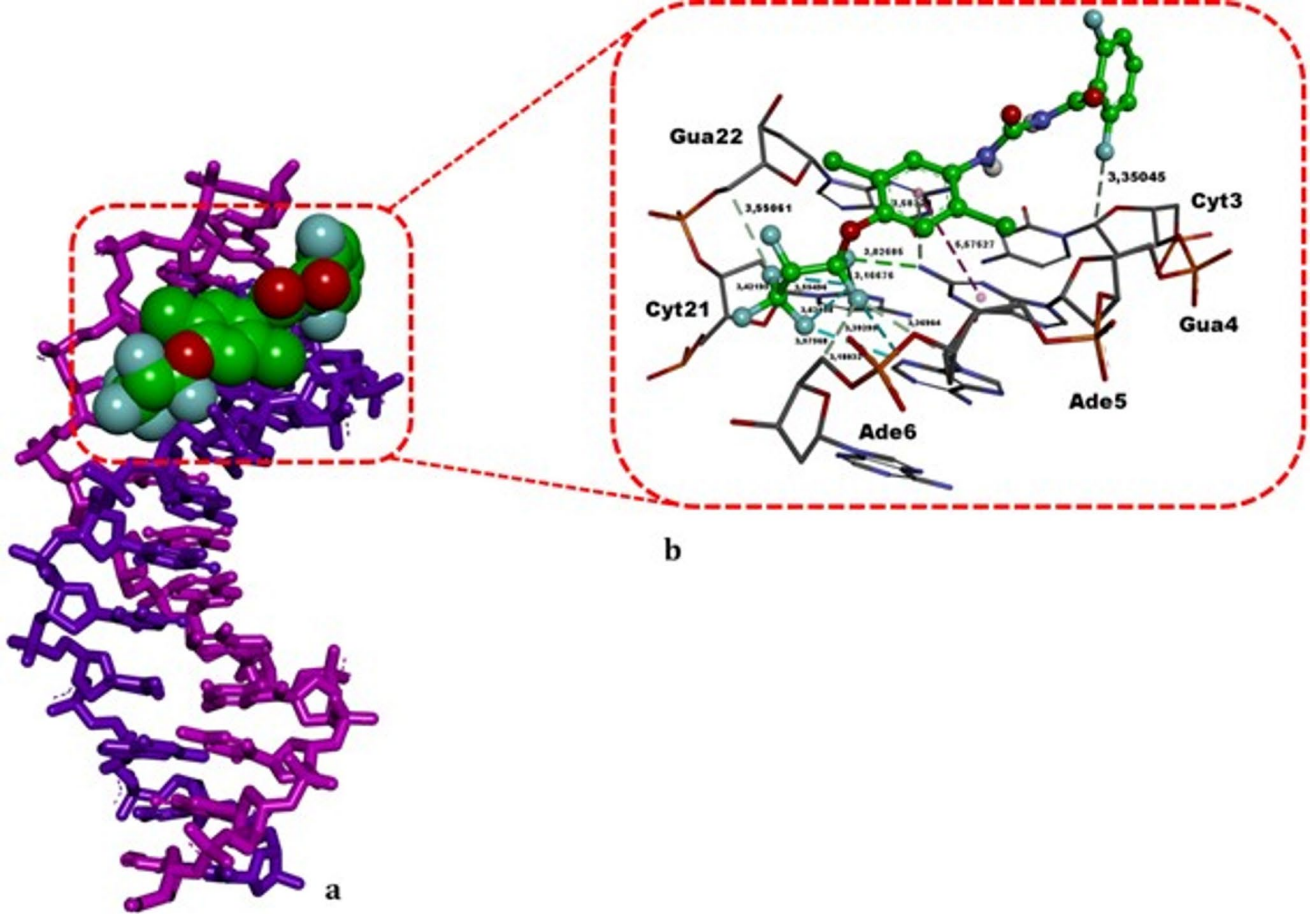
Binding mode of lufenuron with the double-stranded DNA fragment. **a** Three-dimensional representation illustrating lufenuron’s recognition within the DNA minor groove; the DNA is displayed in stick format, whereas lufenuron is shown in CPK mode. Note that, lufenuron is precisely fit within the minor groove. **b** Magnified interaction diagram detailing lufenuron’s contacts with adjacent DNA nucleotides. Green and light green dashed lines indicate hydrogen bonding, aqua dashed lines denote halogen interactions, and a light purple dashed line represents hydrophobic contact. Bold black annotations indicate the non-bonded interaction distances (in Å) and the base names. All structural modeling and visualization were carried out using DS Studio v16

The post-docking analysis of lufenuron with B-DNA (Table 7; Fig. 11) reveals that the ligand adopts a minor groove binding mode. It forms a classical hydrogen bond with Gua4 (3.03 Å) and multiple non-classical hydrogen bonds with Cyt3 (3.35 Å), Gua4 (3.58 Å), Ade5 (3.37 Å), Ade6 (3.18 Å), Cyt21 (3.42 Å), and Gua22 (3.55 Å). Additionally, lufenuron engages in a π–π T-shaped hydrophobic interaction with Gua4 (5.58 Å) and exhibits halogen interactions with Ade5 (3.39 Å, 3.58 Å) and Cyt21 (3.17 Å, 3.55 Å, 3.62 Å). With a binding energy of − 8.59 kcal/mol, lufenuron’s molecular affinity is significantly higher than that of the experimental mutagen MMS (ΔG = − 3.04 kcal/mol). Thus, lufenuron’s binding interactions with B-DNA—characterized by hydrogen bonds and halogen interactions with both GC and AT bases—demonstrate a versatile minor groove recognition pattern. In conclusion, experimental data and computational docking suggest that lufenuron may exhibit a bimodal mechanism of cytogenotoxic effects in *A. cepa* root cells through both stable DNA binding and considerable CYP81A12 and CYP81A21 blocking. In parallel with enzymatic inhibition, the stable association of lufenuron within the DNA minor groove may further exacerbate genotoxic stress by interfering with DNA integrity or repair processes. Together, impaired detoxification via CYP81A inhibition and direct DNA interactions provide a biologically plausible framework for the accumulation-mediated cyto-genotoxic effects detected in *A. cepa* root cells. Such accumulation-mediated stress is also known to promote oxidative imbalance, which may further amplify DNA damage and trigger downstream cell death pathways.

**Table 7:** Predicted Docking energies (kcal/mol), binding conformations, and DNA interaction results (including bond distances in Å) of Methyl methanesulfonate and Lufenuron

Compound									Miscellaneous
	Receptor	ΔG_best_ (kcal/mol)	Binding mode	Classical H-bond	Non-classical H-bond	Hydrophobic (Pi-Pi T-shaped)	Halogen (fluorine)	Pi-sulfur	
MMS (positive control)	B-DNA	-3.04	Minor groove	—	Ade6 (3.36 Å, 3.64 Å), Cyt21 (3.27 Å), Gua22 (3.40 Å)	—	—	Ade5 (5.80 Å), Cyt21 (5.93 Å)	
Lufenuron	B-DNA	-8.59	Minor groove	Gua4 (3.03 Å)	Cyt3 (3.35 Å), Gua4 (3.58 Å), Ade5 (3.37 Å), Ade6 (3.18 Å), Cyt21 (3.42 Å), Gua22 (3.55 Å)	Gua4 (5.58 Å)	Ade5 (3.39 Å, 3.58 Å), Cyt21 (3.17 Å, 3.55 Å, 3.62 Å)	—	

MMS: methyl methanesulfonate – experimental positive control

Δ*G*_best_: Binding free energy (*kcal/mol*) of the most favorable docking pose.

## Conclusion

The findings of this study indicate that lufenuron exerts significant cytogenotoxic effects on *A. cepa* root tip cells, as reflected by increased DNA damage, various CAs, inhibited root growth, reduced MI, cell cycle arrest in the subG1 phase, and alterations in total RAPD profiles, including both the appearance and disappearance of bands. Molecular docking results showed that lufenuron forms a stable binding complex with CYP81A12 and CYP81A21 enzymes. Additionally, lufenuron displayed energetically favorable interactions with GC-rich regions of dsDNA. However, these interactions should be regarded as supportive and hypothesis-generating rather than as direct experimental evidence of causality. Overall, lufenuron appears to act as a multimodal cytotoxic agent in the meristematic cells of *A. cepa*. Nonetheless, further investigations are essential to fully elucidate the molecular mechanisms underlying lufenuron-induced cytogenotoxicity.

## Data Availability

Data will be made available on the reader’s request.
